# Possible mechanism underlying the effect of *Heshouwuyin*, a tonifying kidney herb, on sperm quality in aging rats

**DOI:** 10.1186/1472-6882-14-250

**Published:** 2014-07-17

**Authors:** Siyun Niu, Jingbo Chen, Fei Duan, Qingliang Song, Mingyue Qin, Zhenshan Wang, Jingze Liu

**Affiliations:** 1 School of Life Science, Hebei Normal University, Shijiazhuang 050024, Hebei Province, China; 2 School of Basic Medicine, Hebei University, Baoding 071000, Hebei Province, China; 3 College of Life Science, Hebei University, Baoding 071002, Hebei Province, China

**Keywords:** Heshouwuyin, StAR, P450scc, Sperm quality, Leydig cells

## Abstract

**Background:**

Herb mixtures are used as alternatives to hormone therapy in China for the treatment of partial androgen deficiency in aging men. However, the compositions of these herb mixtures are complex and their mechanisms are often unknown. This study investigates the effect of Heshouwuyin, a Chinese herbal compound for invigorating the kidney, on the control of testosterone secretion and sperm function.

**Methods:**

Aged Wistar rats were administered with Heshouwuyin. A Shouwu pill group and young group were used as controls.

**Results:**

Morphology, chemiluminescence, fluorescence immunohistochemistry, and western blot showed that the epididymal sperm of naturally aged rats had intact plasma membranes. They also had abnormal mitochondrial function and DNA integrity, a significant decline in serum testosterone levels, and significant pathological changes in the structure of testicular tissues. Heshouwuyin significantly improved sperm function and serum testosterone levels, and improved testicular morphology. Moreover, the curative efficacy of Heshouwuyin after 60 days was better than that of Heshouwuyin after 30 days and the Shouwu pill group.

**Conclusion:**

Heshouwuyin exerts an important role in controlling testosterone secretion and sperm function.

## Background

Currently, both the average quantity and quality of sperm in men is languishing, and the situation is worsening [1]. From 1940 to 1990, the average sperm count decreased 45%, from 113 × 10^6^/mL to 66 × 10^6^/mL. Meanwhile, average semen volume decreased to 2.75 mL from 3.4 mL [1]. The minimum reference value of male sperm count decreased to 15 × 10^6^/mL from 60 × 10^6^/mL in 1940s [2,3]. Sex hormones have regulatory effects on the physiological function of the testes [4]. Testosterone, secreted by Leydig cells within the testes, is a sex hormone required for the production of sperm. With decreasing testosterone secretory volume, sperm productivity declines, gonad function is weakened, and sexual dysfunction might result [5]. Studies indicate that the serum free testosterone of males declines with age after 30 years old, with an annual decline of 1–2% after 40 [6]. In a male population south of San Diego, total testosterone, free testosterone, and available testosterone have been found to respectively decrease 3 ng/dL, 0.1 ng/dL, and 2.86 ng/dL yearly, whereas androgen-binding protein increase 0.41 nmol/L yearly. Furthermore, testosterone bioavailability drops 1% annually with age [7]. Although sex hormones are used to combat the effects of aging in clinic, they increase the probability of breast cancer, endometrial cancer, prostate cancer, and include other side effects [8].

Traditional Chinese medicine has unique effects on aging with increased secretion of sex hormones. Heshouwuyin is made up of Shouwu pills, *Epimedium*, *Salvia miltiorrhiza*, and *Wolfiporia extensa*. Previous findings showed that Heshouwuyin can improve antioxidant capacity and dyslipidemia, and regulate p53/pRb in the gonadal cell senescence pathway of D-galactose induced aging rats [9,10]. In addition, Heshouwuyin can significantly improve epidermal growth factor and its receptor in the testes of D-galactose induced aging rats. Heshouwuyin can also regulate hypothalamic-pituitary-testicular secretion of GnRH, gonadotropin, insulin-like growth factor-1, and testosterone. The establishment of the D-galactose induced aging rat model is based on metabolic theory, but aging is the result of many factors working together. Naturally aged rats exhibit a true manifestation of aging and are therefore more useful. Our previous research has shown that Heshouwuyin can increase the concentration of testosterone [11], but its mechanism and influence on sperm quality are not clear. Therefore, we used naturally aged rats subjected to prophylactic treatment of Heshouwuyin to test their sperm quality, testicular tissue morphology, and changes in testosterone levels. We also created a testicular Leydig cell-aging model by *in vitro* primary culture to analyze the effects of Heshouwuyin on testosterone secretion. More specifically, we aimed to study Leydig cells’ ability to increase testosterone secretion, and to clarify the mechanisms underlying the regulatory effect of Heshouwuyin on sperm quality in aging rats.

## Methods

### Design

A randomized controlled animal and cell experiment.

### Time and setting

The experiment was completed at School of Life Science, Hebei Normal University, and School of Basic Medicine, Hebei University, from April, 2011 to March, 2013.

### Animals

Ninety-five male Wistar rats, clean grade, weighing 350–390 g, were provided by the Experimental Animal Laboratory, Quality Inspection Center of Shandong Lukang Pharmaceutical Group Co., Ltd., P. R. China (license No. 20080001). Disposal of experimental animals was performed in accordance with the *Guidance Suggestions for the Care and Use of Laboratory Animals*, formulated by the Ministry of Science and Technology of China [12]. The experimental procedures were conducted according to the guidelines by the Animal Care and Ethics Committee of Hebei Normal University and Hebei University, P. R. China.

### Quantitative analysis of experimental animals

Ninety-five Wistar rats, 12 months old, were randomly divided into five groups after 1 week adaptive feeding. A young control group (YCG, n = 10), 12 months, and a natural aging group (NAG, n = 15), 18 months, were both intragastrically administered normal saline. The Heshouwuyin group 1 (SWY1G, n = 15), 16 months after birth, was given Heshouwuyin (4.8 g/100 g body weight) intragastrically for 60 days. The Heshouwuyin group 2 (SWY2G, n = 15), 17 months after birth, was given Heshouwuyin (4.8 g/100 g body weight) intragastrically for 30 days. The Shouwu pill control group (SWPG, n = 15), 16 months after birth, was given a Heshouwu pill intragastric (4.12 g/100 g body weight) suspension for 60 days. In the two Heshouwuyin groups, the anti-aging effects of different drug administration times were observed. In addition, the anti-aging effects of Heshouwuyin and Shouwu pills were compared. After removal of rats suffering from cancer or other diseases, 10 rats in each group were included in this study.

### Chinese herbal compound preparation

The Heshouwuyin prescription used is composed of: *Polygonum multiflorum*, *Cistanche deserticola*, *Radix Achyranthis Bidentatae*, *Epimedium*, *Salvia miltiorrhiza*, and *Poria cocos*. All herbs were purchased from Hebei Hospital of Traditional Chinese Medicine. Herbs were cut into pieces and mixed with the mass ratio of 3:2:3:2:5:3, respectively. The mixture was immersed in distilled water that was eight times the mass of the mixture for 1 hour, decocted with water twice (once for 30 minutes), and then filtered and concentrated. The final concentration of 4.8 g/mL was stored at 4°C until use. The herbal compound was re-warmed to 25–30°C before administration.

The Shouwu pill prescription used is composed of: *Polygonum multiflorum*, *Cistanche deserticola*, and *Radix Achyranthis Bidentatae*, which were all purchased from Hebei Hospital of Traditional Chinese Medicine. Herbs were cut into pieces and mixed at the mass ratio of 3:2:3, respectively. The mixture was immersed in distilled water that was eight times the mass of the mixture for 1 hour, decocted with water twice (once for 30 minutes), and then filtered and concentrated. The final concentration of 4.12 g/mL was stored at 4°C until use. The herbal compound was re-warmed to 25–30°C before administration.

According to the adult dose conversion [13], 100 g decocted Heshouwuyin containing 2.4 g crude drug was equivalent to the adult dosage. Our preliminary findings implicated that twice the amount of adult dosage produced the best effects. Therefore, 100 g Heshouwuyin containing 4.8 g of crude drug was considered the administration dosage. The adult dose of Shouwu pills was 2.06 g/mL. In the present study, the dosage of Shouwu pills was 4.12 g/mL, which was twice the adult dose. Rats were intragastrically administered at a dose of 0.4 mL/100 g body weight, at 15:00 daily, for 60 days or 30 days.

### Serum preparation

The prepared Heshouwuyin and Shouwu pills were heated to 25–30°C before administration. Wistar rats, weighing 350 g, were selected and subjected to intragastric administration of Heshouwuyin or Shouwu pills, once a day, for 7 days. At the end of the 7^th^ day, 6% chloral hydrate was used to anesthetize rats 1 hour after administration, and blood samples were collected from the rat’s heart aseptically.

### Collection of sperm cells and testicular tissue

After weighing, the rats were anesthetized using 6% chloral hydrate (0.5 mL/100 g body weight). Then, a capillary pipet was inserted into the post-inner canthus venous plexus to collect 2 mL of blood. After centrifugation at 1000 rpm for 10 minutes, the supernatant was extracted for determination of testosterone levels. With 75% ethanol disinfection, the abdominal cavity was opened to remove the caudal epididymis at room temperature followed by two rinses with normal saline at 37°C. Then, the caudal epididymis was placed in a frozen pipe filled with 1 mL 37°C normal saline, cut three times, and incubated in a 37°C water bath for 30 minutes until the sperm swam out. The bilateral testicular tissues were quickly removed, filtered, and weighed using an electronic analytical balance (ESJ200-4, Shanghai, China) for testicle parameter calculation. The testicular parameter was calculated with the following formula: testicular parameter = testicular wet weight / body weight × 100%.

### Observation of the areas of seminiferous tubes and layers of seminiferous epithelium

Testes were removed and fixed for 24 hours at room temperature in 0.1 M pH 7.4 phosphate buffer and 4% paraformaldehyde. Samples were dehydrated with an ethanol and toluene series and embedded in paraffin. Serial sections (4 μm) were mounted on gelatin-coated glass slides and stained with hematoxylin and eosin [14]. The layers of seminiferous epithelium in 100 seminiferous tubule walls were counted under high magnification (400×) with a light microscope (Leica DM 6000 M, Germany), the areas of seminiferous tubes were measured through planimetry with image analysis software (Image Pro Plus 6.0). Mark the outline along with the tubule boundary by mouse after opening view image, then the software can automatically calculate pixel area values of the marked outline [15,16]. By importing the scale factor of pixels distance in the image and the actual distance, the area of seminiferous tubules will be obtained. Three sections were analyzed from each animal.

### Biochemical luminescence determination of testosterone levels

An automated chemiluminescent microparticle immunoassay system (Beckman Unicel D × I 800, USA) was used to determine the testosterone levels using a testosterone assay kit (Beckman Coulter Inc., P33560). The procedures were performed in strict accordance with the kit instructions. The testosterone levels were automatically detected by the instrument in ng/mL.

### Sperm quality investigation

For determining the integrity of the plasma membrane, 1 mg fluorescein diacetate (FDA), (purchased from Fanbo Biochemicals Co. Ltd., Beijing, China, P22020), was dissolved in 1 mL acetone (stored in darkness) and propidium iodide (PI) (purchased from Sigma, St. Louis, USA, P8080). 0.1 M phosphate buffered saline (PBS) solution was used to prepare 400 μg/mL PI solution that was kept in darkness at 4°C. The newly collected sperm suspension, 0.5 mL, was centrifuged at 1000 rpm at room temperature using a super centrifuge (Eppendorf 5415D, Germany) for 2 minutes to remove the supernatant. After washing with 0.1 M PBS twice, the sperm concentration was adjusted to 5 × 10^6^/mL. Then, FDA solution was added to a final concentration of 20 μg/mL for staining at 37°C. Afterwards, PI solution was added to a final concentration of 5 μg/mL, for 3 minute staining. Under a fluorescence microscope (OLYMPUS E53, Japan), 200 sperms were counted to calculate the percentage of sperms with green fluorescence. Green fluorescence indicated a sperm with an intact plasma membrane, while red fluorescence indicated a sperm with a damaged plasma membrane.

To study the sperm mitochondrial function, 0.5 mL of the newly collected sperm suspension was centrifuged at 1000 rpm for 2 minutes to remove the supernatant. Following two washes with 0.1 M PBS, the sample was centrifuged again to remove the supernatant. 100 μL of 20 μg/mL rhodamine 123 (Sigma, USA, R8030) was added to prepare a 400 μg/mL stock solution using PBS. Before use, the stock solution was diluted to 20 μg/mL using PBS (stored at 4°C in darkness). After staining for 30 minutes at 37°C in darkness, the diluted solution was adjusted to the sperm concentration of 5 × 10^6^/mL using 0.1 M PBS. Using fluorescence microscopy, green fluorescent sperms had normal mitochondrial function, and weakened green fluorescence or sub-section phenomenon indicated decreased mitochondrial function. A total of 200 sperms were counted to calculate the percentage of green fluorescent sperms.

To detect sperm DNA integrity, the acridine orange fluorescence detection kit (GMS14018.1v.A; GENMED, Shanghai, China) was used. The newly collected sperm suspension 0.5 mL was placed into a 1.5 mL Eppendorf tube, and centrifuged at 1000 rpm for 2 minutes to remove the supernatant. After washing twice with 1 mL cleaning solution A, the sample was centrifuged to remove the supernatant. Then, the sperm concentration was adjusted to 5 × 10^6^/mL. Smears were prepared and dried naturally. After that, 200 μL fixative solution B was added for fixation at room temperature for 2 hours, and the residual fixative was dried using filter papers. Staining solution C, 200 μL, was added for staining in the darkness at room temperature for 5 minutes. Finally, 200 μL cleaning solution A was used to wash twice or three times, and filter paper was used to dry. Green fluorescence indicated sperms with normal DNA, while red or yellow fluorescence indicated those with abnormal DNA. A total of 200 sperms were counted to calculate the percentage of green fluorescent sperms.

### Isolation, culture, and identification of Leydig cells in the testes

According to the differential centrifugation adherent method, juvenile male Wistar rats, aged 10–20 days, were sacrificed for removal of bilateral testes under sterile conditions. The outer fascia and blood vessels were peeled from the testicular tissue on ice, placed into a 50 mL centrifuge tube, and digested using 0.05% collagenase I (twice the amount of the testicular tissue). When the testicular tissue was removed and the seminiferous tubules unraveled into strips without breaking, an equal volume of Dulbecco’s modified Eagle’s medium/Ham’s nutrient mixture F-12 (DMEM/F12) medium containing 10% fetal bovine serum was added to terminate the digestion, and was left to stand 2 minutes. The solution was filtered with 200-mesh stainless steel sieve and placed into another 50 mL centrifuge tube. The filtrate was centrifuged at 1500 rpm for 10 minutes to remove the supernatant. Then, the specimen was resuspended with 0.1 M PBS and centrifuged at 1000 rpm for 5 minutes. After removal of the supernatant, 4 mL DMEM/F12 medium containing 10% fetal bovine serum, l00 U/mL penicillin, and l00 U/mL streptomycin. The centrifuge tube was placed in an incubator at 37°C, 5% CO_2_ for 2 hours. Cells were removed and the medium was aspirated. Cells were rinsed three times with 0.1 M PBS to eliminate miscellaneous cells after testicular Leydig cells were adherent. Finally, the cells were cultured in 4 mL DMEM/F12 containing 10% fetal bovine serum. After 21 hours of primary culture, the medium was removed from the six-well plates, and 3β-hydroxysteroid dehydrogenase was added, 2 mL per well. Then, the culture plates were incubated overnight. On the next day, the cytoplasm of positive cells was gray under the microscope. The positive rate was over 95%.

### Leydig cell aging models

H_2_O_2_ and FeSO_4_ solutions, at a final concentration of 50 μM and 100 μM, respectively, were added into the six-well culture plates containing Leydig cells cultured for 48 hours. After culture 8 hours, H_2_O_2_ and FeSO_4_ were removed. Then, the cells were placed in DMEM/F12 containing 10% fetal bovine serum for 72 hours. β-galactosidase, a cell senescence marker [17], had a higher level in the natural aging group than the young control group, indication of the Leydig cell aging model was successfully established. Then, the Leydig cells were divided into five groups. For the normal control group, Leydig cells were cultured in DMEM/F12 medium for 128 hours. For the Heshouwuyin control group, Leydig cells were cultured in DMEM/F12 medium for 48 hours followed by culture in 10% Heshouwuyin-containing serum for another 80 hours. Then, the toxicity of Heshouwuyin on Leydig cells was detected with reference to the normal control group. For the Leydig cell aging group, 50 μM H_2_O_2_ and 100 μM FeSO_4_ solutions were added for 8 hours to Leydig cells that had been cultured for 48 hours in the incubator. Then, after removal of H_2_O_2_ and FeSO_4_, the cells were placed in serum-containing DMEM/F12 for 72 hours. For the Heshouwuyin intervention group, after culture in DMEM/F12 for 48 hours, Leydig cells were cultured with 50 μM H_2_O_2_ and 100 μM FeSO_4_ for 8 hours. Then, after removal of H_2_O_2_ and FeSO_4_, cells were cultured in DMEM/F12 medium containing 10% Heshouwuyin-containing serum for 72 hours. The protective effect of Heshouwuyin on the aging of Leydig cells in the testes was observed.

### β-galactosidase enzyme assay

The β-galactosidase staining kit was used, which purchased from Genmed Scientifics Inc., USA(GMS10010.1). According to the experimental requirements, the Leydig cells were cultured primarily using Petri dishes. After the culture medium was aspirated, the cells were washed twice with 0.1 M PBS, and fixed using 1 mL β-galactosidase fixative for 15 minutes. Then, the fixative was aspirated, and the cells were washed with 0.1 M PBS three times for 3 minutes each. β-galactosidase dye working solution was added, 1 mL per well, along with 10 μL A solution, 10 μL B solution, 930 μL C solution, and 50 μL X-Gal solution. Cells were incubated at 37°C overnight. The six-well plates were covered with plastic wrap to prevent evaporation. Then, the working fluid was removed. Under the optical microscope (Leica DM 6000 M, Germany), the cytoplasm of β-galactosidase positive cells was blue. The number of blue cells out of 200 cells was observed and recorded. The experiments were repeated five times for robust statistical analysis.

### Fluorescence immunocytochemistry for observation of StAR and P450scc expression

Sterilized 20 mm coverslips were placed in 90 mm Petri dishes. The cells were seeded in the Petri dishes at density of 2 × 10^4^/mL. The corresponding treatment was given in each group. Samples were fixed in 4% paraformaldehyde for 30 minutes, incubated in 0.5% Triton X-100 for 20 minutes, and cultured in 3% H_2_O_2_ for 15 minutes to eliminate endogenous peroxidase activity. The samples were rinsed with 0.1 M PBS three times for 2 minutes each before each experimental procedure. After rinsing with 0.1 M PBS three times for 2 minutes each, normal goat serum was added, and the samples were inoculated at 37°C for 30 minutes. Filter paper was used to absorb the serum. Diluted rabbit anti-StAR, P450scc monoclonal antibody (1:100; Santa Cruz Biotechnology Inc., Santa Cruz, CA, USA) was added and incubated with samples at 4°C overnight. Then, samples were rinsed with PBS three times, for 5 minutes each. Fluorescent dye-labeled secondary antibody working solution (goat anti-rabbit IgG, Santa Cruz) was added and incubated with samples at 37°C in darkness for 30 minutes. Green granules in the cytoplasm were observed under the fluorescence microscope, and the number of positive cells out of 200 cells was recorded. The experiments were repeated five times for robust statistical analysis.

### Western blot assay for determination of StAR and P450scc protein expression in Leydig cells

Cultured cells in each group were digested with 0.25% trypsin and centrifuged to remove the supernatant. The total protein of each sample was quantified by a BCA protein assay kit. In each group, 50 μg Leydig cell proteins were mixed with loading buffer, placed in a boiling water bath for 5–10 minutes and cooled naturally. Proteins were electrophoretically separated at 80 V in the stacking gel and at 100 V in the separating gel, and transferred to a polyvinylidene difluoride (PVDF) membrane overnight at 4°C with a constant 30 V. The film was set and sealed in 5% skim milk powder, and gently shaken at room temperature for 1 hour. The dried PVDF membrane was placed into a hybridization bag, and incubated in Tris-buffered saline with Tween 20 (TBST) containing rabbit anti-StAR, P450scc, and β-actin monoclonal antibodies (antibody dilution: 1:200; Sigma) at room temperature for 2 hours. After rinsing with TBST three times (for 10 minutes per session), the membrane was gently shaken using a water bath shaker. After drying and placing into the hybridization bag, the membrane was cultured in TBST containing horseradish peroxidase-labeled corresponding secondary antibody (goat anti-rabbit IgG, 1:2,000, Santa Cruz) at 37°C for 1 hour. Then, the membrane was rinsed with TBST three times, for 10 minutes per session, and colored. Pierce chemiluminescent substrates, solution A and solution B, were mixed at a ratio of 1:1 for coloration at room temperature for 5 minutes, then the reaction mixture was aspirated, and the fluorescence/chemiluminescence imaging system (Fuji Film, Tokyo, Japan) was used for analysis. The intensity of each band was quantified using density analysis software (AlphaEase FC software, Miami, FL, USA). The relative expression of P450scc and StAR protein was the density ratio of each band and β-actin in each experiment.

### Statistical analysis

Data are expressed as mean ± SD, and analyzed using SPSS 16.0 statistical software (SPSS, Chicago, IL, USA). Normal distribution was assessed by qq plots and variance homogeneity by robust variance test. The data showed a normal distribution and homogeneity of variance. Differences between groups were compared using one-way analysis of variance. Intergroup comparison was done using SNK-q test. A value of *P* < 0.05 was considered statistically significant.

## Results

### Testicular tissue evaluation

**Figure 1:**
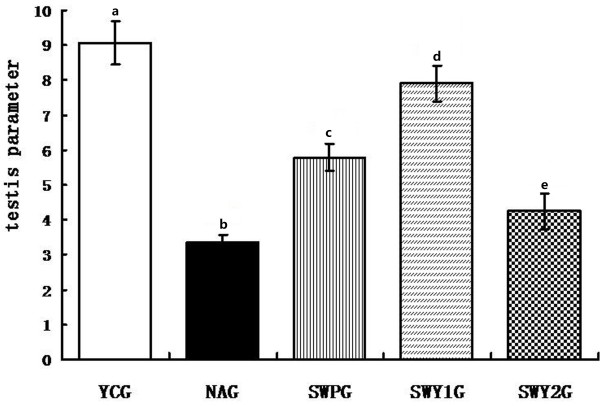
**Testicular parameter changes in different groups.** YCG: young control group; NAG: natural aging group; SWPG: Shouwu pill group; SWY1G: Heshouwuyin group 1; and SWY2G: Heshouwuyin group 2. Data are expressed as the mean ± SD (n = 10 in each group). Columns with different letters represent statistically different values, while same letters indicates no significant difference.

For evaluate of testicular tissue, the testicular parameter, the average area of seminiferous tubules and the number of seminiferous epithelium was measured. The testicular parameter in the natural aging group was significantly lower than that in the young control group (*P* < 0.01). However, the testicular parameter in the two Heshouwuyin groups and Shouwu pill group, especially in Heshouwuyin group 1, was significantly higher than that in the natural aging group (*P* < 0.01). The testicular parameter in the Heshouwuyin group 1 was significantly higher than that in the Shouwu pill group (*P* < 0.01; Figure 1). The highest testicular parameter was found in the Heshouwuyin group 1.

**Figure 2:**
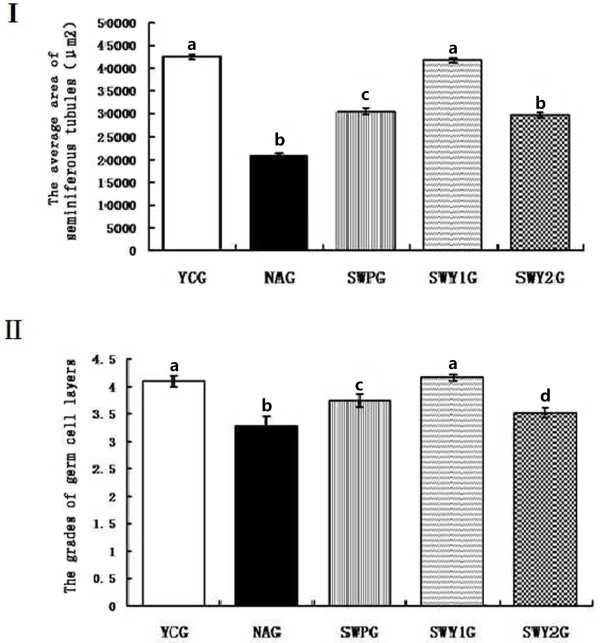
**The average area of seminiferous tubules (I) and number of seminiferous epithelium (II) in each group.** YCG: young control group; NAG: natural aging group; SWPG: Shouwu pill group; SWY1G: Heshouwuy in group 1; and SWY2G: Heshouwuyin group 2. Each histogram represents the mean ± SD of the values for 3 rats. Columns with different letters represent statistically different values, while same letters indicates no significant difference.

The average area of seminiferous tubules and the number of seminiferous epithelium were significantly lower in the natural aging group than that in the young control group (*P* < 0.05). Compared with the natural aging group, the average area of seminiferous tubules and the number of seminiferous epithelium were significantly higher in all three drug treatment groups (*P* < 0.05). In addition, the average area of seminiferous tubules and the number of seminiferous epithelium were significantly higher in the Heshouwuyin group 1 than that in the Shouwu pill group and Heshouwuyin group 2 (*P* < 0.05; Figure 2).

### Epididymal sperm quality

**Figure 3:**
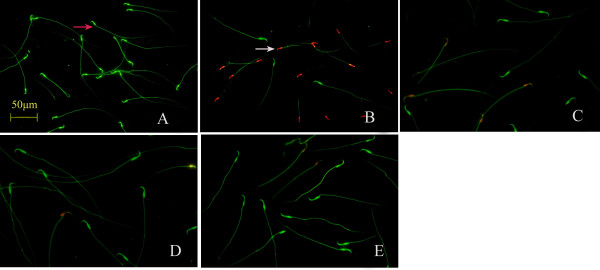
**Fluorescence staining showing sperm plasma membrane integrity in each group.** The green fluorescence indicates the intact plasma membrane (red arrow), and the red or yellow fluorescence reflects damaged plasma membrane (white arrow). The number of sperm cells with the intact plasma membranes was significantly lower in the natural aging group **(B)**, while the number of sperm cells with intact plasma membranes was higher in the young control group **(A)**, the Shouwu pill group **(D)**, Heshouwuyin group 1 **(E)** and Heshouwuyin group 2 **(C)**, compared with the natural aging group. Scale bar, 50 μm.

**Figure 4:**
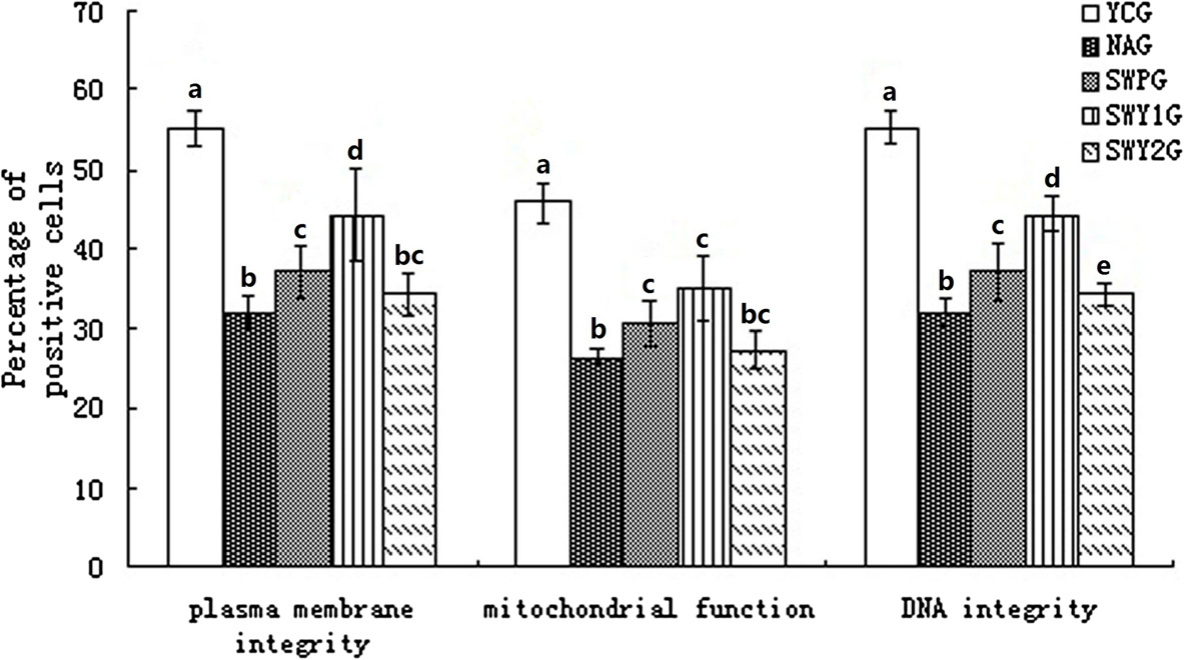
**Plasma membrane integrity, mitochondrial function and DNA integrity of sperm in different groups.** YCG: young control group; NAG: natural aging group; SWPG: Shouwu pill group; SWY1G: Heshouwuyin group 1; and SWY2G: Heshouwuyin group 2. Each histogram represents the mean ± SD of the values for 10 rats. Columns with different letters represent statistically different values, while same letters indicates no significant difference.

According to the literature [18], the combination of FDA and PI staining can be used to observe the integrity of the plasma membrane (Figure 3). Our results showed that the number of sperm cells with green fluorescence was significantly lower in the natural aging group than that in the young control group (*P* < 0.01). The number of sperm cells with green fluorescence was significantly higher in the two Heshouwuyin groups and the Shouwu pill group that that in the natural aging group (*P* < 0.01). Compared with the Shouwu pill group, the number of sperm cells with green fluorescence was significantly higher in the Heshouwuyin group 1 (*P* < 0.01; Figure 4). Furthermore, the highest number of sperm cells with green fluorescence was found in the Heshouwuyin group 1.

**Figure 5:**
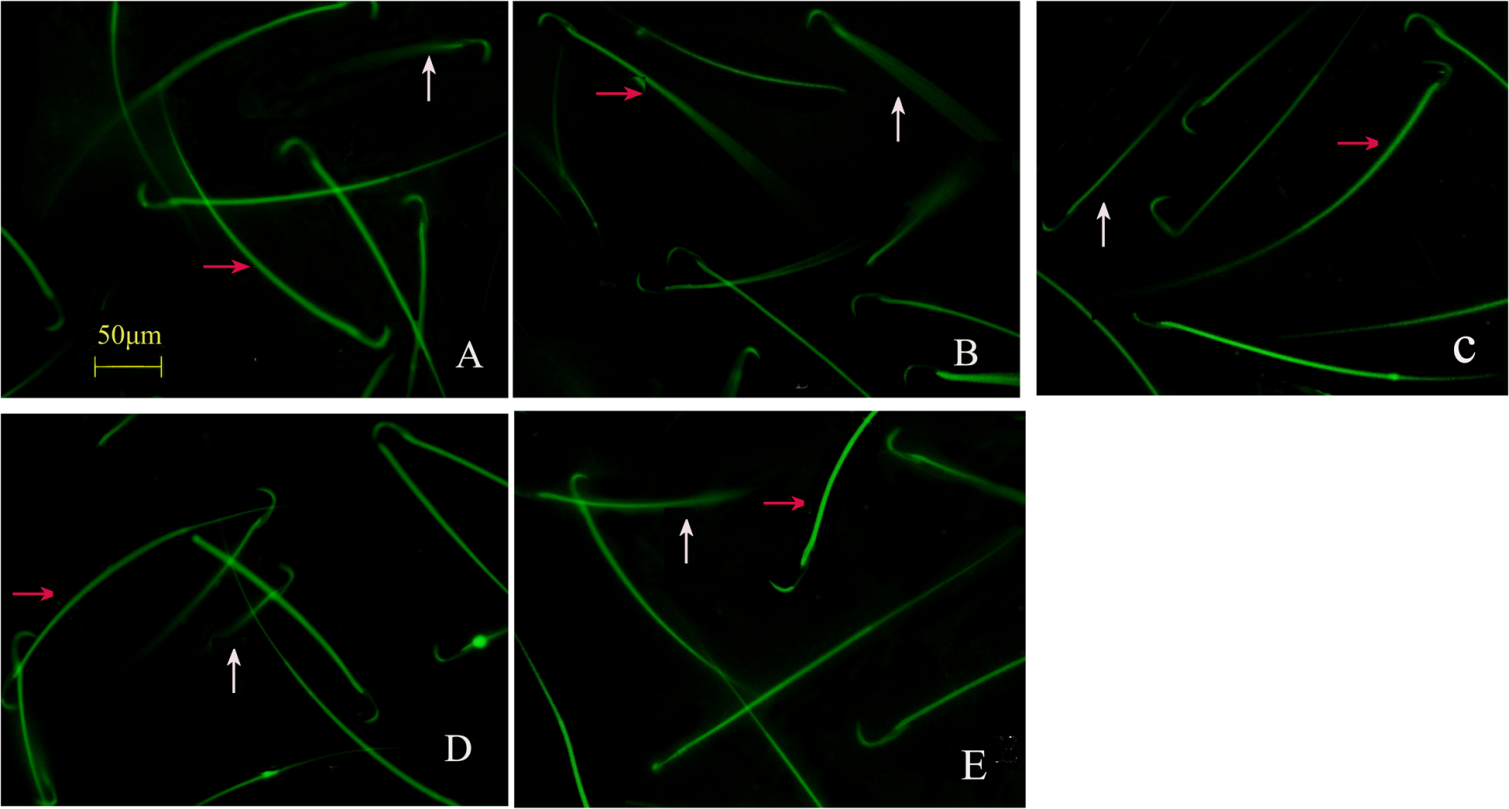
**Fluorescence staining showing sperm mitochondrial function in each group.** The sperm cells with normal mitochondrial function exhibited strong green fluorescence (red arrow), those with decreased mitochondrial function appeared to have weakened green fluorescence or sub-section phenomenon. The number of sperm cells with green fluorescence was significantly lower in the natural aging group **(B)**, while the number of sperm cells with normal mitochondrial function was higher in the young control group **(A)**, Shouwu pill group **(D)**, Heshouwuyin group 1 **(E)** and Heshouwuyin group 2 **(C)**, compared with the natural aging group. Scale bar, 50 μm.

Under a fluorescence microscope after Rhodamine 123 staining, sperm cells with normal mitochondrial function show strong green fluorescence, while those with decreased mitochondrial function have weakened green fluorescence or sub-section phenomenon (Figure 5) [19]. We found that the number of sperm cells with green fluorescence was significantly lower in the natural aging group than that in the young control group (*P* < 0.01). The number of sperm cells with green fluorescence was significantly higher in the Heshouwuyin group 1 and the Shouwu pill group than that with the natural aging group (*P* < 0.01). However, there was no difference in the number of sperm cells with green fluorescence between the Heshouwuyin group 1 and the Shouwu pill group (*P* > 0.05, Figure 4).

**Figure 6:**
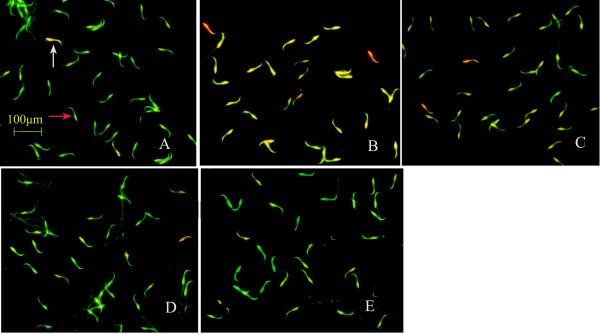
**Fluorescence staining showing sperm DNA integrity in each group.** The sperm head was fluorescent green indicating normal DNA (red arrow), and red or yellow fluorescence reflecting to abnormal DNA (red arrow). The number of sperm cells with normal DNA was significantly lower in the natural aging group **(B)**, while the number of sperm cells with normal DNA was higher in the young control group **(A)**, the Shouwu pill group **(D)**, Heshouwuyin group 1 **(E)**, and Heshouwuyin group 2 **(C)**, compared with the natural aging group. Scale bar, 100 μm.

Sperm DNA integrity was tested using acridine orange fluorescence detection kit [20]. Normal sperm heads were fluorescent green indicating normal DNA, but red or yellow fluorescent sperm heads indicated abnormal DNA (Figure 6). The number of sperm cells with normal DNA was significantly lower in the natural aging group and the number of sperm cells with abnormal DNA was significantly higher than that in the young control group (*P* < 0.01). Compared with the natural aging group, the number of sperm cells with normal DNA was higher, while the number of sperm cells with abnormal DNA was significantly lower in the Heshouwuyin and Shouwu pill groups (*P* < 0.01). Compared with the Shouwu pill group, the number of sperm cells with normal DNA was significantly higher in the Heshouwuyin group 1 (*P* < 0.01). The highest number of sperm cells with normal DNA was found in the 60-day Heshouwuyin group (Figure 4).

### Serum testosterone levels

**Figure 7:**
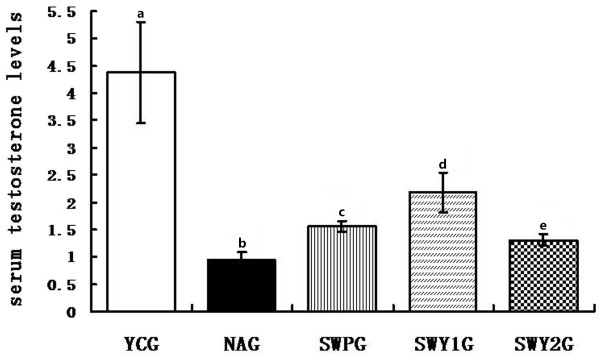
**Concentration of serum testosterone in different experimental groups.** YCG: young control group; NAG: natural aging group; SWPG: Shouwu pill group; SWY1G: Heshouwuyin group 1; and SWY2G: Heshouwuyin group 2. Each histogram represents the mean ± SD of the values for 10 rats. Columns with different letters represent statistically different values, while same letters indicates no significant difference.

Biochemical luminescence assay was performed to detect serum testosterone levels. The serum testosterone level in the natural aging group was significantly lower than that in the young control group (*P* < 0.01). Compared with the natural aging group, two Heshouwuyin groups and Shouwu pills groups had higher serum testosterone levels (*P* < 0.01). The serum testosterone level in the Heshouwuyin group 1 was significantly higher than that the Shouwu pills group (*P* < 0.01). The 60-day Heshouwuyin group showed a better effect on the serum testosterone levels than that in the other groups (Figure 7).

### β-galactosidase in Leydig cells

**Figure 8:**
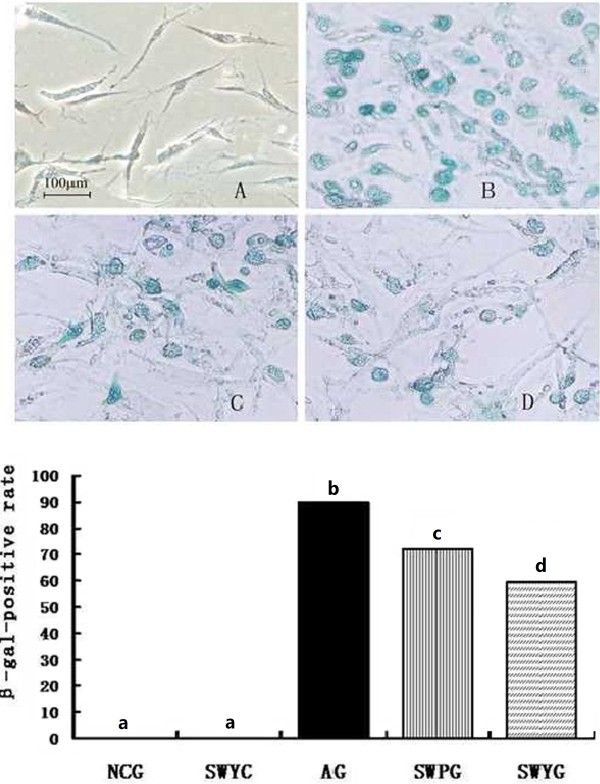
**Expression of LC β-galactosidase in different experimental groups.** Upper panel: Illumination of expression of LC β-galactosidase in different experimental groups. The cytoplasm of positive cells was blue, the normal control group **(A)** had some blue-stained cells, but there were significantly more blue-stained cells in the aging group **(B)**. The β-galactosidase-positive rate in the aging group was significantly higher than that in the Shouwu pill group **(C)** and Heshouwuyin group **(D)**. Scale bar, 100 μm. Lower panel: Relative expression of LC β-galactosidase in different experimental groups. NCG: normal control group; SWYC: Heshouwuyin control group; AG: aging group; SWPG: Shouwu pill group; SWYG: Heshouwuyin group. Each histogram represents the mean ± SD of the values for 5 rats. Columns with different letters represent statistically different values, while same letters indicates no significant difference.

β-galactosidase is located in the cytoplasm, and cells positive for β-galactosidase are blue in color. The β-galactosidase-positive rate in the Leydig cell aging group was significantly higher than that in the normal control group (*P* < 0.05). β-galactosidase was lower in the Heshouwuyin control group than that the aging group (*P* < 0.05). The expression of β-galactosidase in Leydig cells was lower after treatment with Heshouwuyin and Shouwu pills as compared with the aging group (*P* < 0.05). β-galactosidase was lower in the Heshouwuyin group than that in the Shouwu pill group (*P* < 0.05). However, there was no difference in the expression of β-galactosidase in Leydig cells between the normal control group and Heshouwuyin groups (*P* > 0.05). β-galactosidase expression contributed to determining the state of cell aging, and Heshouwuyin treatment could significantly delay the aging of Leydig cells (Figure 8).

### Testosterone levels in Leydig cell supernatant

**Figure 9:**
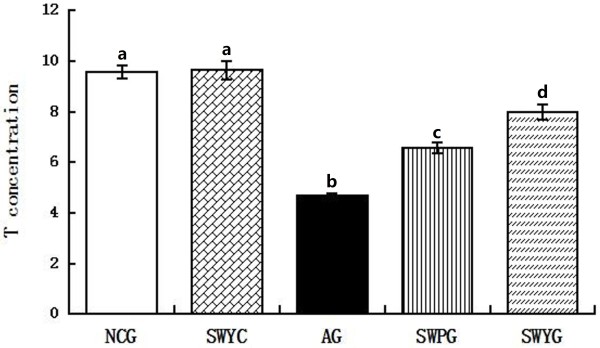
**Concentration of Leydig cell testosterone in different experimental groups.** NCG: normal control group; SWYC: Heshouwuyin control group; AG: aging group; SWPG: Shouwu pill group; SWYG: Heshouwuyin group. Each histogram represents the mean ± SD of the values for 5 rats. Columns with different letters represent statistically different values, while same letters indicates no significant difference.

We used the biochemical luminescence method to detect the testosterone levels in the supernatant of cultured Leydig cells. The testosterone level in the Leydig cell aging group was significantly lower than that in the normal control group and Heshouwuyin control group (*P* < 0.05), while there was no difference between the latter two groups. Heshouwuyin and Shouwu pill intervention could increase testosterone levels to varying degrees, as compared with the Leydig cell aging group (*P* < 0.05). Moreover, the Heshouwuyin group testosterone level significantly higher than that in the Shouwu pill group (*P* < 0.05; Figure 9).

### StAR and P450scc in Leydig cells

**Figure 10:**
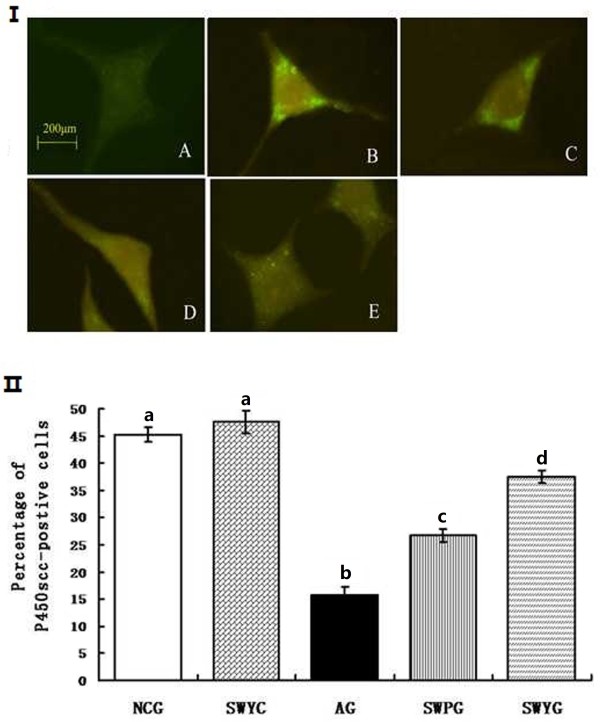
**Fluorescence immunocytochemistry for observation of P450scc expression (I) and P450scc-positive cells (II) in Leydig cells. A**: aging group; **B**: normal control group; **C**: Heshouwuyin control group; **D**: Shouwu pill group; **E**: Heshouwuyin group. Scale bar, 200 μm. NCG: normal control group; SWYC: Heshouwuyin control group; AG: aging group; SWPG: Shouwu pill group; SWYG: Heshouwuyin group. Each histogram represents the mean ± SD of the values for 5 rats. Columns with different letters represent statistically different values, while same letters indicates no significant difference.

**Figure 11:**
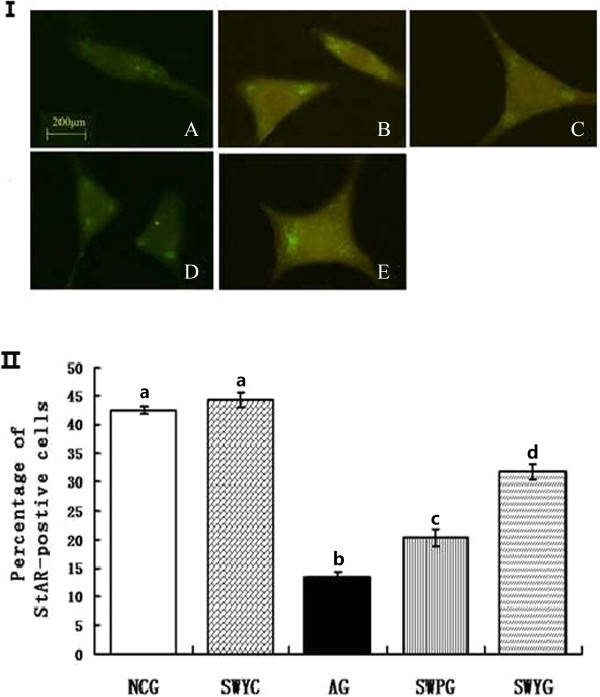
**Fluorescence immunocytochemistry for observation of StAR expression (I) and StAR-positive cells (II) in Leydig cells. A**: aging group; **B**: normal control group; **C**: Heshouwuyin control group; **D**: Shouwu pill group; **E**: Heshouwuyin group. Scale bar, 200 μm. NCG: normal control group; SWYC: Heshouwuyin control group; AG: aging group; SWPG: Shouwu pill group; SWYG: Heshouwuyin group. Each histogram represents the mean ± SD of the values for 5 rats. Columns with different letters represent statistically different values, while same letters indicates no significant difference.

**Figure 12:**
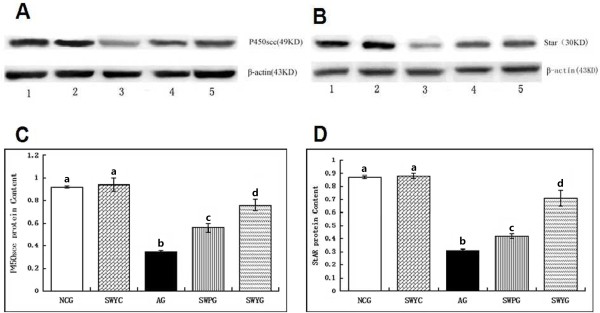
**Western blot assay for determination of P450scc (A, C) and StAR (B, D) protein expression in Leydig cells.** 1: normal control group; 2: Heshouwuyin control group; 3: aging group; 4: Shouwu pill group; 5: Heshouwuyin group. NCG: normal control group; SWYC: Heshouwuyin control group; AG: aging group; SWPG: Shouwu pill group; SWYG: Heshouwuyin group. Each histogram represents the mean ± SD of the values for 5 rats. Columns with different letters represent statistically different values, while same letters indicates no significant difference.

Immunofluorescence staining showed that positive signals of steroidogenic acute regulatory protein (StAR) and cytochrome P450 cholesterol side (P450scc) were located in the cytoplasm of Leydig cells, and the positive products were brownish green granules (Figures 10 and 11). In the aging group, the percentage of StAR and P450scc positive cells was significantly lower than that in the normal control group (*P* < 0.05). There were significantly more positive StAR and P450scc cells in the Heshouwuyin control group, Heshouwuyin and Shouwu pill groups than those in the aging group (*P* < 0.05). Moreover, the Heshouwuyin group had higher percentage of StAR and P450scc positive cells than those the Shouwu pill group (*P* < 0.05, Figures 10 and 11). Semi-quantitative analysis showed that in the aging group, the expressions of StAR and P450scc protein in the Leydig cells were significantly lower than those in the normal control group (*P* < 0.05). The expression of StAR and P450scc was higher in the Heshouwuyin control group, Heshouwuyin and Shouwu pill groups than that in the aging group (*P* < 0.05 ). However, there was no difference between the Heshouwuyin and normal control groups (*P* > 0.05). Moreover, the Heshouwuyin group had higher expressions of StAR and P450scc than those in the Shouwu pill group (*P* < 0.05, Figure 12). StAR and P450scc are key proteins to the testosterone synthesis in Leydig cells. Heshouwuyin could increase the expression of these two proteins, increase testosterone synthesis, and thereby improve the quality of sperm.

## Discussion

Heshouwuyin is made up of Shouwu pills, *Epimedium*, *Salvia miltiorrhiza*, and *Wolfiporia extensa*. We found that Heshouwuyin exhibited anti-aging and sperm-protective effects. In fact, anti-aging and reproductive function improvement have been reported for several constituents of Heshouwuyin. Pharmacological studies have indicated that *Epimedium* posses hormone-like effects. It promotes the synthesis of sex hormones to slow the degenerative changes in testicular tissue by increasing testosterone synthesis enzyme activity [21]. In addition, *Epimedium* improves the function of rat Leydig cells *in vitro* and promotes the basal secretion of Leydig cells [22]. Cistanche glycosides eliminate free radicals and protect hydroxyl-induced DNA damage [23]. *Salvia miltiorrhiza*, known as Danshen in Chinese traditional medicine, is effective at promoting blood circulation and removing (or decreasing) blood stasis. Water-soluble components of *Salvia miltiorrhiza* can improve blood parameters in elderly guinea pigs, reduce blood viscosity, and significantly enhance oxygen transport capacity [24]. Water-soluble extract components of *Wolfiporia extensa* may retard aging by increasing the content of skin hydroxyproline [25].

In the present study, we explored the effect of Heshouwuyin on sperm quality and testosterone secretion from Leydig cells. By observing the integrity of the epididymal sperm plasma membrane, mitochondrial function, and DNA integrity, we found that naturally aged rats appeared to have abnormal sperm quality and a significant decrease in serum testosterone levels. The pathological changes in the testicular tissue structure were consistent with previous findings. Heshouwuyin treatment promoted significantly higher sperm quality and serum testosterone levels, and showed better testis morphology than the aging controls. Moreover, the 60-day Heshouwuyin intervention was better than the 30-day Heshouwuyin and Shouwu pill interventions. Therefore, Heshouwuyin has an important regulatory role in testosterone secretion and sperm function.

The effects of Heshouwuyin on StAR and P450scc expression, and testosterone synthesis in Leydig cells are unclear. Oxidative stress theory is now a more accepted hypothesis of aging [26]. Oxidative stress-induced premature senescence is commonly used in aging studies *in vitro*. Sustained stress can induce DNA damage, which can lead to changes in gene structure and function, changes in gene expression, and cell cycle arrest, thereby ultimately resulting in cell senescence [27,28]. In this study, we used H_2_O_2_ and FeSO_4_ to build an aging model of Leydig cells. Interaction between H_2_O_2_ and FeSO_4_ resulted in the production of hydroxyl radicals, and oxidized hydroxyl radicals can induce cell senescence. However, this effect has not been reported in aging models of Leydig cells. After primary culture, Leydig cells were added into H_2_O_2_ and FeSO_4_ at final concentrations of 50 μM and 100 μM, respectively, for 8 hours. By observing β-galactosidase expression and testosterone secretion in Leydig cells, we determined whether the Leydig cell-aging model was successfully established. Our data showed that, after treatment with 50 μM H_2_O_2_ and 100 μM FeSO_4_ for 8 hours, primarily cultured Leydig cells had a higher β-galactosidase-positive rate that in the normal control group. Furthermore, the biochemical luminescence assay showed lower testosterone level in the supernatant of cultured Leydig cells, thus confirming the success of this method to establish a testicular Leydig cell-aging model. Then, we observed StAR and P450scc protein expression in Leydig cells, and found that StAR and P450sc protein expression in the aging group was significantly lower than that in the normal control group, and the secretion of testosterone was also significantly lower in the aging group. However, the Heshouwuyin group had higher StAR and P450sc protein expression and secretion of testosterone in Leydig cells than that in the aging cells. Therefore, Heshouwuyin not only improves testosterone secretion in testicular Leydig cells, but regulates StAR and P450scc. These findings provide valuable evidence for the clinical application of kidney-replenishing drugs to improve partial androgen deficiency in aging males and quality of sperm. However, Heshouwuyin may be associated with side effects including liver abnormalities. More detailed studies on the side effects of Heshouwuyin need to be performed.

The secretion of testosterone decreases obviously with male aging. Decreases in testosterone lead to conditions such as weakness, fatigue, insomnia, depression, irritability, mood swings, loss of libido, erectile dysfunction, loss weight, and osteoporosis, so there is a significant reduction in quality of life [29]. Supplementary methods have been used *in vitro* to slow the clinical symptoms, but there are significant side effects [8]. Heshouwuyin can improve sperm quality and secretion of testosterone, thereby exerting anti-aging effects and improving male reproductive function.

## Conclusions

The present study reveals that Heshouwuyin, a Chinese herbal compound for invigorating the kidney, exerts an important role in controlling testosterone secretion and sperm function. Furthermore, the data provide a more extensive theoretical and experimental basis to better understanding the molecular mechanisms underlying the regulation of testicular reproductive function after Heshouwuyin treatment.

## Abbreviations

FDA: Fluorescein diacetate; PI: Propidium iodide; DMEM/F12: Dulbecco’s modified Eagle’s medium/Ham’s nutrient mixture F-12; StAR: Steroidogenic acute regulatory protein; P450scc: Cytochrome P450 cholesterol side; HCG: Human chorionic gonadotropin; PBS: Phosphate buffered saline; TBST: Tris buffered saline with Tween 20.

## Competing interests

The authors declare that they have no competing interests.

## Authors’ contributions

SYN, ZSW, and JZL designed the study and wrote the paper. JBC, FD, QLS, and MYQ performed the research and analyzed the data. All authors read and approved the final manuscript.

## Pre-publication history

The pre-publication history for this paper can be accessed here:

http://www.biomedcentral.com/1472-6882/14/250/prepub
